# Evaluation of the lithium resource in the Smackover Formation brines of southern Arkansas using machine learning

**DOI:** 10.1126/sciadv.adp8149

**Published:** 2024-09-27

**Authors:** Katherine J. Knierim, Madalyn S. Blondes, Andrew Masterson, Philip Freeman, Bonnie McDevitt, Amanda Herzberg, Peng Li, Ciara Mills, Colin Doolan, Aaron M. Jubb, Scott M. Ausbrooks, Jessica Chenault

**Affiliations:** ^1^ US Geological Survey, Lower Mississippi-Gulf Water Science Center, Nashville, TN, USA.; ^2^ US Geological Survey, Geology, Energy & Minerals Science Center, Reston, VA, USA.; ^3^ Arkansas Department of Energy and Environment, Office of the State Geologist, Little Rock, AR, USA.

## Abstract

Global demand for lithium, the primary component of lithium-ion batteries, greatly exceeds known supplies, and this imbalance is expected to increase as the world transitions away from fossil fuel energy sources. High concentrations of lithium in brines have been observed in the Smackover Formation in southern Arkansas (>400 milligrams per liter). We used published and newly collected brine lithium concentration data to train a random forest machine-learning model using geologic, geochemical, and temperature explanatory variables and create a map of predicted lithium concentrations in Smackover Formation brines across southern Arkansas. Using these predicted lithium maps with reservoir parameters and geologic information, we calculated that there are 5.1 to 19 million tons of lithium in Smackover Formation brines in southern Arkansas, which represents 35 to 136% of the current US lithium resource estimate. Based on these calculations, in 2022, 5000 tons of dissolved lithium were brought to the surface within brines as waste streams of the oil, gas, and bromine industries.

## Teaser

Smackover Formation brines in southern Arkansas may contain 5.1 to 19 million tons of lithium based on machine learning.

## INTRODUCTION

**Fig. 1.**
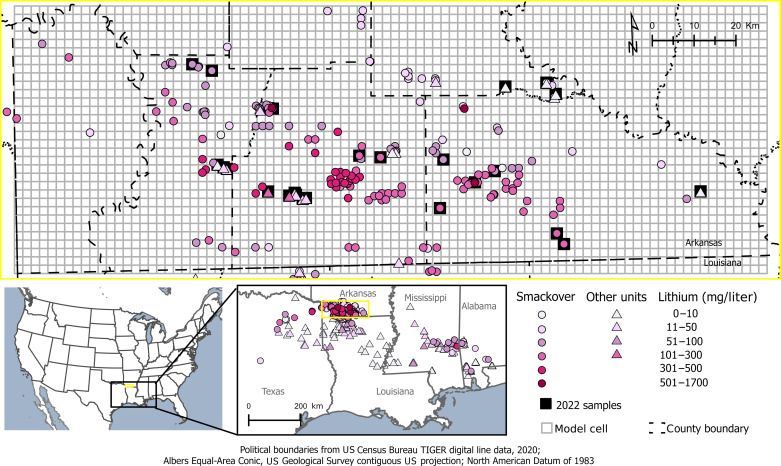
Map showing observed lithium concentrations in brines in the Gulf Coast region. Lithium concentrations shown for brines from the Smackover Formation and other geologic units in southern Arkansas and the Gulf Coast region. Three lithium concentrations of >500 mg/liter from southern Arkansas are shown for reference but were not used for summary statistics or modeling.

Lithium is identified as a critical mineral because of its use in batteries and the growing importance of transitioning from fossil fuel–driven internal combustion engines to electric and hybrid vehicles (*1*). As of 2023, commercial-scale lithium production in the US was only from Nevada and Utah (*2*), but extensive lithium deposits occur throughout the US (*3*). The Upper Jurassic (Oxfordian) Smackover Formation (hereafter referred to as the Smackover or Smackover Formation) is an example of a laterally extensive petroleum and oilfield brine system in the Gulf of Mexico region that includes locally high concentrations of bromide (>5000 mg/liter) and lithium (>300 mg/liter), especially in southern Arkansas (*3*–*5*) (Fig. 1). Projects are ongoing to extract lithium from these Smackover Formation brines, in some cases leveraging the wastewater from commercial bromine production (*6*). Thus, the Smackover Formation in southern Arkansas represents a potentially important region of US lithium production to meet the demands of a growing market. In addition, lithium in brines co-produced during oil and gas production represents an opportunity to extract a commodity from what is otherwise a waste stream (*7*).

Basin and oilfield brines have received increasing attention for lithium exploration and extraction (*7*–*10*), especially in the past decade as lithium production has increased (*2*) and future demand is forecast to increase substantially (*11*). Oilfield brines may be an important lithium resource because these brines occur globally, are otherwise considered a waste product from the oil, gas, and brine industries, and—depending on the viability of direct lithium extraction technologies—would not require a large footprint similar to the evaporative processes used to concentrate basin brines (*7*, *12*). Despite the global prevalence of oilfield brines, lithium concentrations and correlations with geologic features and other geochemical constituents vary within and across basins (*4*, *9*, *13*, *14*). For example, lithium in brines is found to generally increase with total dissolved solids (*9*) and depth (*14*–*16*). Lithium mass yields were found to vary across the Marcellus Shale in Pennsylvania, based on variable concentration and volumes of produced waters (*13*). Within the Gulf Coast of the US, lithium in Smackover Formation brines was found to correlate with potassium, possibly from interaction with feldspathic minerals or clays (*15*, *17*). These assessments of lithium provide insights into the variability, geochemical relations, and possible sources of lithium in oilfield brines. Despite these general relations, using an average concentration from brine samples for resource assessments may not fully capture the spatial variability of lithium across basins, and quantifying this variability is important for commercial lithium extraction (*7*).

In recent decades, machine learning has become an important tool to characterize the spatial variability of geochemical constituents in subsurface waters (*18*) as the algorithms can identify complex and nonlinear patterns and handle large and diverse explanatory variable datasets (*19*). The machine-learning models can incorporate mapped geologic explanatory variables, thus providing the models with information about geologic features that may be important predictors of groundwater chemistry (*18*, *20*). Using machine-learning models to map mineral prospectivity is an emerging field (*21*), but similar research to predict shallow groundwater chemistry using machine learning has shown the ability to produce accurate maps of aquifer chemistry (*18*, *20*, *22*). The goals of this work were to generate a map of predicted lithium concentrations using machine learning—specifically random forest (RF)—and use the predictions with reservoir and geologic characteristics to determine the mass of lithium in Smackover Formation brines across southern Arkansas.

Lithium in Smackover Formation brines has been evaluated across southern Arkansas, but a spatially continuous prediction of lithium in subsurface brines and estimate of lithium mass have not been completed. Individual Smackover Formation fields within southern Arkansas have been assessed for commercial lithium production (*23*, *24*), and the area has been included as part of a regional lithium economic assessment (*25*). Machine learning has been used to create spatially continuous predictions of lithium concentrations in drinking water across the US but at much shallower depths than Smackover brines (*18*). In addition, a classification machine-learning model was used to investigate how brine geochemistry can be used to predict lithium in the Smackover Formation, but this investigation only predicted lithium at brine sample locations (*17*). In this study, we use published and newly collected brine lithium concentration data to train a machine-learning model and create a spatially continuous map of predicted lithium in Smackover Formation brines across southern Arkansas using geologic, geochemical, and temperature explanatory variables. The predicted lithium concentrations can be used with geologic and reservoir characteristics—such as formation thickness, porosity, and water-to-oil ratios—to quantify the mass of lithium in brines. Although the focus of this study is the Smackover Formation, brine samples were also collected from units overlying the Smackover Formation, which provides additional context about potential mixing or lithium resources in other geologic formations that have received less attention (*15*).

## RESULTS

**Fig. 2.**
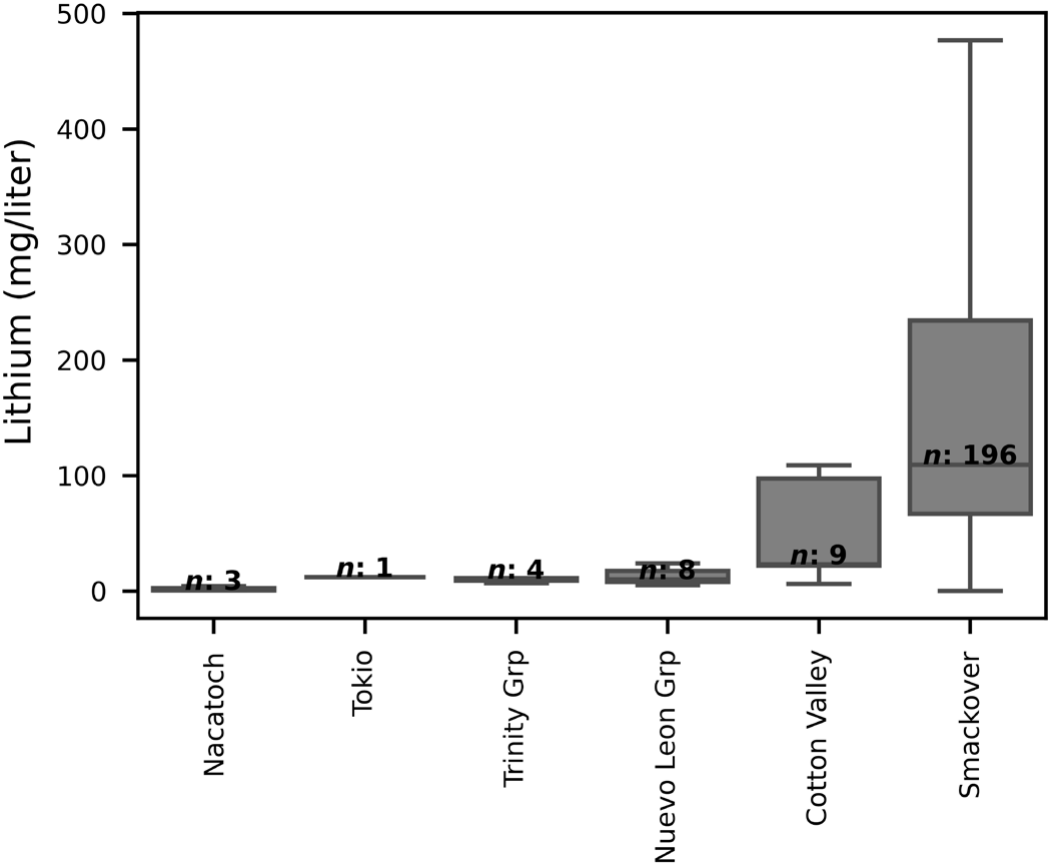
Lithium concentrations in brines by geologic formation or group in the southern Arkansas model domain. Box plots represent the interquartile range, with whiskers extending to points within 1.5 times the interquartile range. For geologic ages and formal names, refer to the main text. Grp, group.

Lithium concentrations ranged from 0.08 to 1700 mg/liter in brines across the Gulf Coast region, but only 3 of 544 samples were >500 mg/liter (Fig. 1). These three samples are associated with US Bureau of Mines samples collected between 1965 and 1970 and compiled in the US Geological Survey’s Produced Waters Geochemical Database (PWGD) version 3.0 (*4*); concentrations of >500 mg/liter could not be verified from source data. In addition, at two of the three locations, Smackover Formation brines were sampled in 2022 from nearby wells (the exact wells could not be resampled), and concentrations were found to be much lower (between 95 and 178 mg/liter). Therefore, lithium concentrations of >500 mg/liter were not used for subsequent modeling or summary statistics. Lithium concentrations within the model domain of southern Arkansas ranged from 0.27 to 477 mg/liter (Table 1 and table S1) and varied by formation (Figs. 1 and 2). Brine samples used in the RF model were collected from 1965 to 2022, with most samples collected in the 1960s and 1970s. The Smackover Formation had the highest lithium concentrations compared to other units, with a median of 110 mg/liter (Fig. 2). Although lithium concentrations were lower in units overlying the Smackover Formation, the Cotton Valley Formation contained elevated lithium concentrations up to approximately 100 mg/liter (Fig. 2 and table S1).

**Table 1.** Observed and predicted lithium concentrations in Smackover Formation brines in southern Arkansas.

Statistic	Observed	Predicted (wells)	Predicted (raster cells)
Size	221	221	2524
Minimum	0.27	3.7	13
Median	98	97	87
Mean	142	141	109
Maximum	477	408	389

**Fig. 3.**
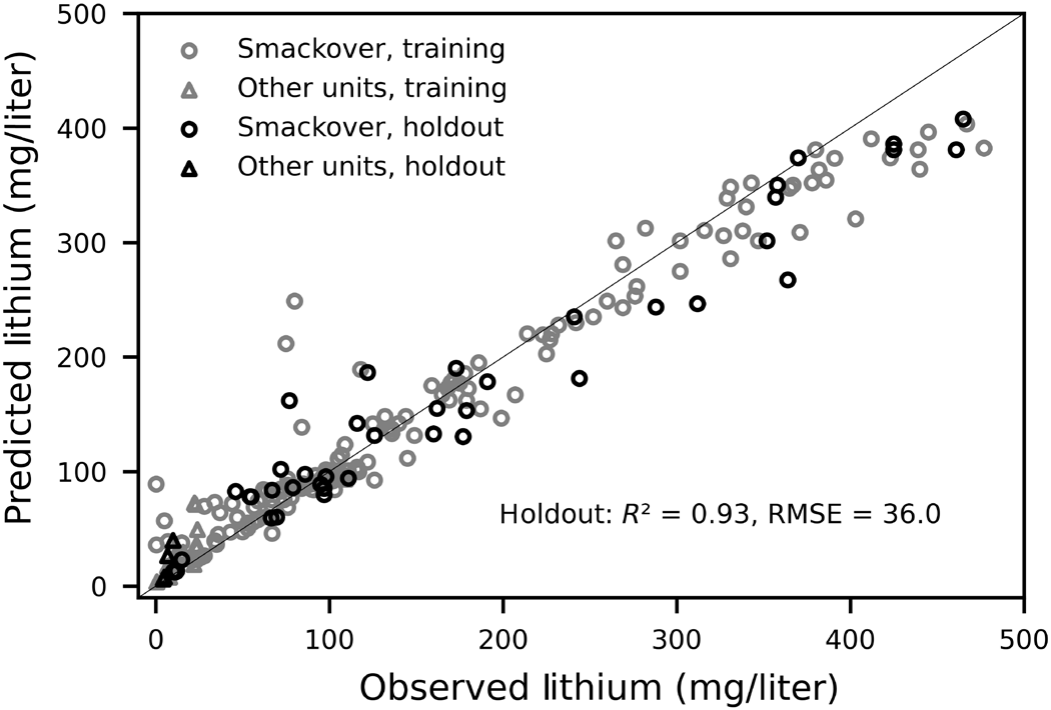
Observed versus predicted lithium concentrations. Data represent lithium concentration data in brines from the Smackover Formation and other geologic units (see main text for details). Training data were used to tune the RF machine-learning model, and holdout data were used to evaluate model performance.

Lithium concentrations predicted by the RF model for all samples in the model domain ranged from 3.7 to 408 mg/liter (Table 1 and Fig. 3). The final RF machine-learning model had hyperparameters of 6 mtry (number of randomly selected explanatory variables), 4 min_n (the minimum number of observations per node), and 200 trees (the number of trees grown); final hyperparameters were found using 10-fold cross-validation tuning on 80% of the data (refer to Materials and Methods for details). Model performance was evaluated using the remaining 20% of the data (holdout data), and the final model had a root mean square error (RMSE) of 36 mg/liter and *R*^2^ of 0.93. High lithium concentrations (>400 mg/liter) were slightly underpredicted (Fig. 3), which is typical of tree-based machine-learning models (*26*). Of concern with the small training dataset is the potential for overfitting, as model accuracy was quite high for both training and holdout data. Cross-validation tuning, evaluating a holdout dataset, and choosing a model within one SE of the model with the lowest RMSE as the final model were all meant to protect against overfitting. One mechanism to evaluate model accuracy would be increasing lithium concentration data available for model training; as of 2023, all available data were used.

**Fig. 4.**
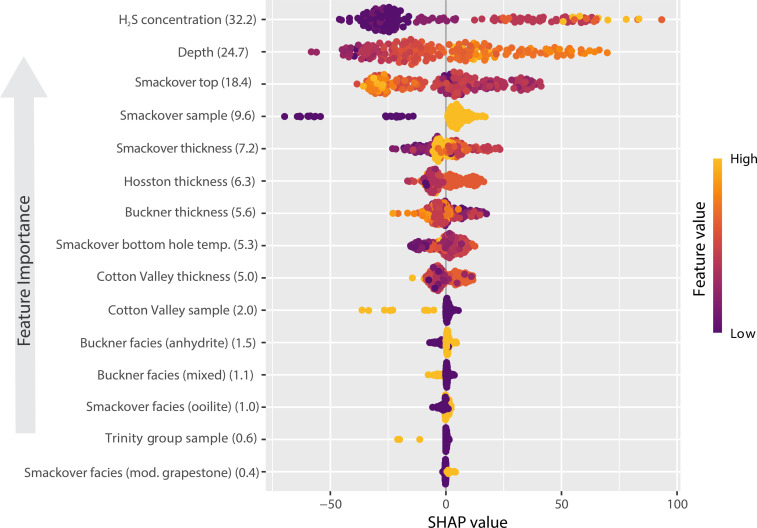
Explanatory variable importance based on calculated SHAP values. The top 15 explanatory variables in the lithium RF machine-learning model, with the importance score shown in parentheses. Higher SHAP values correspond to a higher predicted lithium concentration, and lower SHAP values correspond to a lower predicted lithium concentration. The coloring for each explanatory variable represents the magnitude of that explanatory variable for predicting the corresponding SHAP values.

The top five most important explanatory variables for predicting lithium were dissolved hydrogen sulfide (H_2_S) concentrations in Smackover Formation brines, depth of brine sample, altitude of the top of the Smackover Formation, whether a brine sample was collected from the Smackover Formation or another geologic unit, and thickness of the Smackover Formation (Fig. 4). Detailed digital three-dimensional geologic information mapped across basins is often limited in the public domain; thus, geologic information from historical maps and datasets was digitized and used as explanatory variables for the RF model (refer to the Supplementary Materials). The source of the lithium was not tested with explanatory variables in the RF model. Other geologic information may be important for predicting lithium and can be tested in subsequent modeling efforts. Shapley additive explanation (SHAP) values do provide an explanation that as H_2_S increases, predicted lithium concentration increases (Fig. 4), especially where the Smackover Formation depth is between 2500 and 3000 m deep. Therefore, in southern Arkansas, H_2_S is an important predictor of high lithium concentrations in brines.

**Fig. 5.**
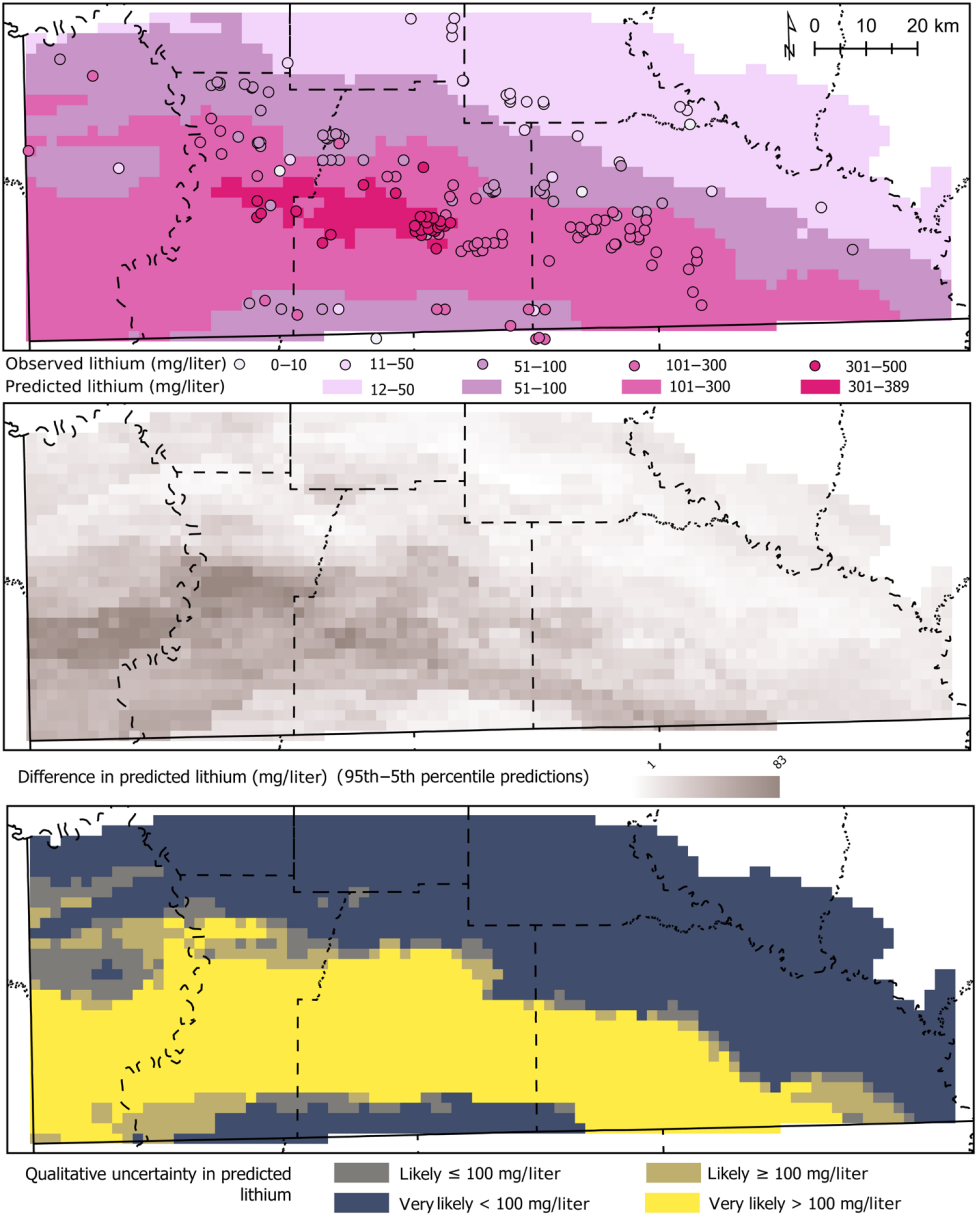
Maps of predicted lithium concentrations and uncertainty. Maps showing spatially continuous predictions of lithium concentrations in Smackover Formation brines compared to observations at wells, range of predicted lithium concentrations between the 95th and 5th percentiles of all one-SE model predictions at each cell, and qualitative uncertainty showing the likelihood of predicted lithium greater or less than 100 mg/liter.

The trained RF model was used to predict lithium at the midpoint altitude of the Reynolds oolite unit of the Smackover Formation at 4-km^2^ resolution (Fig. 5). Predicted lithium concentrations ranged from 13 to 389 mg/liter (Table 1). Lithium could not be predicted in the northeastern part of the model domain where the Smackover Formation facies was calcarenite-carbonate mudstone (*27*) because this facies category was not represented with any lithium observations for RF model training. Predictions were also masked along the northwestern and southern boundaries of the model domain where explanatory variables were missing. The Smackover Formation thins and pinches out to the north, so the prediction maps represent the northern limit of predicting brine chemistry in the Smackover Formation. The model could be extended south and west with additional brine samples and expansion of the geologic framework beyond the current model domain. Explanatory variables were smoothed when resampled to the 4-km^2^ model resolution, such that the holdout accuracy of observed lithium concentrations compared to predictions at the raster cell where the sample was collected was an RMSE of 81 mg/liter.

Uncertainty in RF model predictions—based on the difference between the 95th and 5th percentiles of predictions across all model cells using 425 one-SE models—was 1 to 83 mg/liter (Fig. 5). The model domain used a 4-km^2^ grid cell to balance having as many cells as possible with a lithium concentration sample while minimizing cells with multiple samples. Of the 2054 cells representing the Smackover Formation, approximately 6% included at least one lithium concentration value and 2% included two to eight samples. The SD for cells with multiple samples was <1 to 193 mg/liter, with a median of 11 mg/liter. Therefore, the range of observed lithium concentrations within model cells is on the same order of magnitude as the uncertainty of the RF model predictions based on one-SE models. The uncertainty predictions were used to classify whether a model cell was likely to be greater or less than 100 mg/liter, which is a concentration cutoff used for some direct lithium extraction technologies (*7*). Lithium concentrations were predicted to be very likely >100 mg/liter throughout a broad swath of the Smackover Formation, which also co-occurs with field-sampled brines with observed lithium concentrations of >100 mg/liter (Fig. 5). Within the model domain, we estimate that approximately 42% of the Smackover Formation contains brines with lithium concentrations of >100 mg/liter.

## DISCUSSION

To calculate the volume of Smackover Formation brines, the thickness, porosity, and water-to-oil ratio of the unit must be known. The thickness of the Reynolds oolite unit of the Smackover Formation varied between 0 (pinching out) and over 122 m thick across the model domain (*27*). When sampled to the 4-km^2^ model resolution, the thickness averaged 73 m. Porosity ranged from 0.5 to 29% in three wells in Lafayette County that were sampled between approximately 2499 and 2652 m deep (*n* = 363) (*28*). Porosity was found to be highest in distinct zones that were between 5 m and 15 m thick (*28*). Average reservoir porosity values ranged from 10 to 31% (*29*). To assess the volume of brines within the Smackover Formation, porosity was assumed to be 10, 20, and 30% across the model domain. Water-to-oil ratios in 43 reservoirs producing from the Smackover Formation ranged from 0.11 to 0.44 or between 11 and 44% brine (*29*). When applied to the 4-km^2^ model resolution, 100 cells included water-to-oil ratios of <0.9 where oil reservoirs were located, and all other cells were assigned a water-to-oil ratio of 0.9 (assuming 90% brine). On the basis of these thickness and porosity ranges, brine volume ranged from 65,000 to 200,000 billion liters (410 to 1200 billion barrels). The entirety of the Reynolds oolite unit of the Smackover Formation does not have a single porosity value spatially (*30*) or vertically (*28*), such that mapped porosity would improve brine volume estimates. Lacking such information, the estimated brine volumes provide a first approximation for the region.

Many areas of the model domain have no oil production, and, thus, no co-produced brines, from the Smackover Formation since 1982. Most oil production since 1982 has occurred in the Magnolia field in Columbia County and brine production from two fields in Columbia and Union counties. The lowest nonzero production from a 4-km^2^ area was 1 barrel in 2000, and the highest was 26 million barrels in 2020 (*31*). In total across the model domain, between 56 (1982) and 300 (in 2011) million barrels of brine have been extracted from the Smackover Formation since 1982 (fig. S3). In 2022, brine production ranged from 0 (no production) to 14.7 million barrels across the model domain and totaled 175 million barrels (fig. S3). Southern Arkansas represents an area where lithium in brines of >100 mg/liter occurs and some portion of the brines are brought to the surface as part of existing commercial oil, gas, and brine waste streams.

The mass of predicted lithium in the Reynolds oolite unit of the Smackover Formation ranged from 5.1 to 19 million tons (or 27 to 100 million tons of lithium carbonate equivalent) based on the prediction maps (Fig. 5) and porosity between 10 and 30% (Table 2). If the 5th percentile of predicted lithium concentrations were used (low concentration scenario), then lithium ranged from 5.1 to 15.2 million tons. If the 95th percentile of predicted lithium concentrations were used (high concentration scenario), then lithium ranged from 6.3 to 19 million tons. The range of estimated lithium mass represents uncertainty associated with the 90th percentile prediction interval from the RF machine-learning model and porosity ranging from 10 to 30%. The difference in calculated lithium mass based on the range of porosity values (i.e., 10 to 30%) was greater than the difference between the 5th and 95th percentiles of predicted lithium for each porosity value (i.e., 10, 20, or 30%) (Table 2). For example, assuming a 30% porosity, the difference between the 95th percentile lithium prediction (high concentration model scenario) and 5th percentile lithium prediction (low concentration model scenario) is approximately 3.8 million tons of lithium. In contrast, assuming the median lithium prediction, the difference between 10 and 30% porosity is approximately 11.3 million tons (Table 1). Therefore, better estimates of Smackover Formation porosity could decrease the uncertainty in estimated lithium mass. Porosity measurements from individual wells, formation horizons, or reservoirs must be extrapolated to regional maps for such an estimate to be used across the model domain. Because of how porosity effects the lithium mass calculation, a companion US Geological Survey data release provides Python code to evaluate different porosity scenarios for estimating brine volumes (*32*).

**Table 2.** Estimated mass of lithium in Smackover Formation brines in southern Arkansas. Lithium mass estimates were based on predicted lithium brine concentrations from low (5th percentile), median (50th percentile), and high (95th percentile) RF machine-learning models and a range of porosity values for the Reynolds oolite unit of the Smackover Formation (10, 20, and 30%). The mass of lithium brought to the surface was based on water production volumes in 2022 associated with gas, oil, or brine wells (*31*).

Li model*	Average predicted Li (mg/liter)	Average porosity (%)	Brine volume (liters)	Lithium (tons)	Lithium LCE* (tons)	Lithium, brought to surface (tons)	Proportion lithium brought to surface (%)
Low	96	10	6.5 × 10^13^	5.1 × 10^6^	2.7 × 10^7^	4454	0.09
Median	107	10	6.5 × 10^13^	5.7 × 10^6^	3.0 × 10^7^	4854	0.09
High	119	10	6.5 × 10^13^	6.3 × 10^6^	3.4 × 10^7^	5142	0.08
Low	96	20	1.3 × 10^14^	1.0 × 10^7^	5.4 × 10^7^	4454	0.04
Median	107	20	1.3 × 10^14^	1.1 × 10^7^	6.0 × 10^7^	4854	0.04
High	119	20	1.3 × 10^14^	1.3 × 10^7^	6.8 × 10^7^	5142	0.04
Low	96	30	2.0 × 10^14^	1.5 × 10^7^	8.1 × 10^7^	4454	0.03
Median	107	30	2.0 × 10^14^	1.7 × 10^7^	9.0 × 10^7^	4854	0.03
High	119	30	2.0 × 10^14^	1.9 × 10^7^	1.0 × 10^8^	5142	0.03

*LCE, lithium carbonate equivalent.

The estimated mass of lithium is dependent on the accuracy of the underlying RF model, which requires a training dataset that adequately represents the lithium resource. In addition, RF models are not an appropriate extrapolation tool because an accurate RF model will predict the range of values in the training data (*33*). For the lithium RF model, we relied heavily on historical data (*4*), with newly collected samples in 2022 targeting areas that were previously unsampled or wells with notably high lithium concentrations (*32*). Because machine-learning models are inherently data greedy and computer resource intensive, it is only in the past decades, and even more recently in the past few years, that mapped predictions from machine-learning models are possible (*33*); therefore, reliance on historical data is a requirement. Any lithium exploration efforts will have to rely on such historical data, at least in part. The mapped predictions represent lithium concentrations from 1965 to 2022 and do not consider any changes in lithium concentrations over time, as repeat samples from the same well were not available.

Uncertainty in mapped, predicted lithium concentrations has implications for lithium exploration and commercial extraction. The RF model slightly overpredicted low values and underpredicted high values (Table 1 and Fig. 3), as is typical of tree-based machine-learning algorithms (*26*). Underpredicting high concentrations will be of more interest to industry extracting lithium from oilfield brines, especially since the overpredicted concentrations are generally <50 mg/liter (Fig. 3). Uncertainty in mapped predictions was highest in the western part of the model domain, especially where there were no lithium concentration data (Fig. 5). Mapped predictions of lithium from machine-learning models will benefit from ongoing sampling, especially across areas that have little brine chemistry data. Despite these challenges, RF models represent an important tool to create prediction maps of critical minerals that will provide more reasonable estimates than assuming constant lithium concentrations across whole regions or basins. The RF model provides an estimate of the lithium resource in the Smackover Formation across southern Arkansas and, therefore, may not represent conditions at individual wells. This is especially important given that local-scale variation in lithium concentrations—along with other brine constituents that may interfere with direct lithium extraction technologies—can greatly affect the feasibility of commercial lithium recovery (*7*). In addition, the RF model is only suitable for predicting lithium in brines in the Smackover Formation, as most of the lithium concentration data used to train the model were collected from the Smackover Formation. Notably, the Cotton Valley Formation was found to have lithium concentrations up to approximately 100 mg/liter (Fig. 2), but data were insufficient to predict lithium in Cotton Valley brines.

The 2023 estimated global lithium resource was 105 million tons, of which 14 million tons were estimated in the US (*2*). The US total includes estimates of continental brines such as the Smackover Formation (*2*). Without knowing what proportion of the US total includes the model domain, the lithium resource in the Smackover Formation brines within southern Arkansas would represent approximately 36% to more than the current (2023) US resource estimate (136%). On the basis of the volume of brine extracted in 2022, approximately 5000 tons—or less than 0.1% of the available lithium resource in the Smackover Formation—has been brought to the surface within brines as waste streams of the oil, gas, and bromine industries (Table 1). Assuming 100% extraction efficiency of lithium from the brines, this would cover the estimated US consumption in 2022 (*34*). Predicted lithium concentrations and estimates of lithium mass in the subsurface represent a first approximation of the in-place resource, and future modeling may benefit from including analysis of technical recoverability.

## MATERIALS AND METHODS

### Study design

The mass of available lithium in brines in the Smackover Formation of southern Arkansas was quantified by (i) acquiring lithium concentrations in brines, (ii) predicting a spatially continuous map of lithium concentrations using an RF machine-learning model, and (iii) calculating the mass of lithium in the Smackover Formation and assessing the mass extracted associated with historical oil and brine production. Model resolution was a 2-km by 2-km square (4 km^2^) grid of 90 columns and 32 rows (Fig. 1). A 4-km^2^ resolution was used for the model domain based on the density of lithium brine data compared to the total area of the model domain. Scripts associated with this modeling workflow are available in a US Geological Survey data release (*32*).

### Geologic framework

The southern Arkansas shelf includes Triassic through Cretaceous sedimentary units (fig. S1) deposited in marine, near-shore marine, or coastal settings with the thickness and structure of units controlled by Triassic rifting, subsequent filling of the Gulf of Mexico, and deformation of salt (*35*). The Triassic-age Eagle Mills Formation unconformably overlies late Paleozoic deposits and is composed of fluvial, deltaic, and lacustrine “red beds” containing diabase and basalt sills (*36*). The Jurassic Louann Salt overlies the Eagle Mills Formation and is a relatively pure-phase halite with minor anhydrite (*35*). The Jurassic Norphlet Formation was deposited as a terrigenous siliciclastic unit and is composed of thin shale, sandstone, and gravel deposits in the north-central Gulf of Mexico (*37*). The Jurassic-age Smackover Formation is typically underlain by either the Norphlet Formation or the Louann Salt where the Norphlet is absent. The Smackover Formation is predominantly limestone in southern Arkansas and is divided into two informal members (*38*): an upper oolitic to chalky porous limestone (known informally as the Reynolds oolite) and a lower member composed of dense argillaceous limestone and dark calcareous shale (known informally as the Brown Dense) (*27*). Most petroleum is produced from the Reynolds oolite (*39*). The lower part of the Smackover Formation serves as an effective regional source rock in the onshore interior salt basins in the north central and northeastern Gulf of Mexico (*40*). Approximately 151 fields across southern Arkansas have produced more than 500 million barrels of oil and condensate from the Smackover Formation since production began in 1936 (*41*, *42*). Brine extraction to produce bromine began in 1957 (*43*). The Buckner Member of the Upper Jurassic Haynesville Formation, composed of red shale and white to pink anhydrite, conformably overlies the Smackover Formation. It can be absent locally and may vary in thickness up to 91 m (*35*).

The upper Jurassic to Cretaceous section of the southern Arkansas shelf includes 100 s of meters of units with locally described oil reservoirs often in lenticular sand lenses; here, only the units that have been sampled for lithium concentrations in brines are described. There is a sharp change in lithology, indicative of a disconformity, between the underlying Upper Jurassic Louark Group and the overlying Upper Jurassic to Lower Cretaceous Cotton Valley Group, which is a nearshore red bed facies in southern Arkansas approximately 914 m thick (*38*). The Lower Cretaceous Nuevo Leon Group includes the Hosston Formation (red shale with interbedded lenses of white sandstone) and Sligo Formation (gray to brown shale with lenses of dense gray limestone and sandstone). Locally, the Sligo Formation includes porous oolitic limestone lenses of the Pettet Limestone Member. The Lower Cretaceous Trinity Group is subdivided, in ascending order, into the Pine Island Shale, James Limestone, Rodessa Formation, Ferry Lake Anhydrite, and Mooringsport Formation (*38*); brines from the James Limestone and Rodessa Formation include lithium concentration data. The James Limestone consists of a fossiliferous, dense limestone, and red and gray shale. The Rodessa Formation consists of oolitic and crystalline limestones, lenticular fine-grained sandy limestone, anhydrite, coquinoid limestones, and gray shales and contains several lenses and tongues that are productive oil reservoirs (*44*). Upper Cretaceous units with lithium brine samples include the Tokio Formation [coarse gray and brown cross-bedded quartz and dark gray lignitic fossiliferous clay (*45*)] and the Nacatoch Formation [predominantly sand with interbedded shale, clay, and calcareous deposits (*46*)].

The source of lithium in Smackover Formation brines is not well understood as the brines have a complex geochemistry inherited from initial seawater evaporation and subsequent interaction with rocks during brine emplacement (*5*, *15*, *47*, *48*). Smackover Formation brines initially acquired high salinity from evaporation of Jurassic seawater and expulsion of fluids from the underlying Louann Salt, but bromide and lithium are both enriched relative to seawater. Correlation between lithium and boron, rubidium, and potassium along with strontium isotope signatures that are more radiogenic than Jurassic seawater suggests that the brines interacted with siliciclastic minerals during emplacement (*47*, *48*). Lithium could be sourced from igneous material—diabase dikes and sills, eroded volcaniclastic sediments, or air fall tuff—as volcanism occurred throughout Triassic rifting and deposition of Jurassic units (*5*, *25*, *35*). Regardless of the initial source of lithium, lithium-containing brines migrated to the Smackover Formation and—similar to the oil resource—were trapped by stratigraphic or structural features (*49*).

### Brine data

#### Historical brine dataset

Brine samples from the US Geological Survey’s PWGD version 3.0 were filtered for the Gulf Coast region within the states of Arkansas, Louisiana, Mississippi, Alabama, and Texas to provide regional lithium concentrations (*4*). This dataset was further filtered for the model domain of southern Arkansas, which included 1168 samples of which 193 included a lithium analysis. These data were mostly from research associated with the US Bureau of Mines (*49*), Moldovanyi and Walter (*47*), and Trout (*50*). More recently collected data associated with lithium extraction in southern Arkansas were also available in the PWGD (*4*) associated with ongoing projects to produce lithium commercially (*23*, *24*). Most historical lithium data were collected from the Smackover Formation, but limited data were available from the Tokio Formation, Nuevo Leon Group (Hosston Formation), and Cotton Valley Group (table S1 and fig. S1) (*32*). Because the historical brine data are from multiple sources, analytical methods and detection limits for lithium varied.

#### Brine sampling and analysis

In August 2022, brine samples were collected from 27 oil and brine wells throughout southern Arkansas from Jurassic and Cretaceous formations to provide updated information about lithium concentrations. The focus of the sampling was on the Smackover Formation, but shallower units overlying the Smackover—including the Nacatoch Formation, Trinity Group (Rodessa Formation and James Limestone), Nuevo Leon Group (Hosston Formation), and Cotton Valley Group—were also sampled to provide information about possible brine mixing between units (Fig. 1 and fig. S1) (*32*).

Water sample collection protocols for total dissolved solids, anions, and major and trace cations (including lithium) followed those outlined in Blondes *et al.* (*51*). Samples were collected at the wellhead or separator in 5-gallon carboys. For any well with a known presence of hazardous H_2_S gas, the fluid in the carboy was purged with ultrahigh purity nitrogen (N_2_) gas using a custom sparging apparatus until H_2_S was below detection after turning off the N_2_. The water was then pumped from the carboy through a 0.45-μm filter using a peristaltic pump and stored in nitric acid–washed high density polyethylene bottles that were triple rinsed with sample. The cation aliquots were acidified to a pH < 2 using TraceMetal grade nitric acid. To measure the dissolved sulfide concentration of the samples, 500-ml amber glass bottles containing a known quantity of zinc acetate saturated solution as a fixative were filled with brine directly at the wellhead or separator. These samples were not sparged with N_2_. All samples were stored on ice and shipped to the US Geological Survey in Reston, Virginia for analysis in the BRInE Laboratory (www.usgs.gov/labs/brine-research-instrumentation-and-experimental-laboratory).

Sulfide concentrations were determined on zinc acetate fixed samples using similar methods detailed in Moldovanyi and Walter (*47*). A known volume of the fixed sample was resuspended and injected anoxically into an N_2_-purged reactor containing anoxic 6-N hydrochloric acid (HCl). For all samples containing >1 mg/liter of iron, HCl was prepared with stannous chloride (SnCl_2_) to reduce oxidation artifacts by ferric iron. The resulting H_2_S was purged from the reactors using N_2_ and carried in fresh 0.3 M zinc acetate traps. Product zinc sulfide (ZnS) was converted to silver sulfide (Ag_2_S) by addition of 0.3 M silver nitrate (AgNO_3_). Ag_2_S was rinsed with ammonium hydroxide (NH_4_OH), filtered, dried, and weighed to assess the concentration of sulfide in the original solution. Replicate measurements of standard sulfide solutions, fixed as ZnS, of 10 and 100 mg of S/liter yielded a complete sulfide recovery of ±5%.

### Predicted lithium concentrations

An RF machine-learning model was developed to predict lithium concentrations in Smackover Formation brines throughout southern Arkansas. The model was developed by (i) assigning explanatory variables to brine samples collected at wells, (ii) tuning the RF model to make predictions at wells and assess model performance, (iii) mapping spatially continuous predictions of lithium concentrations across the Reynolds oolite unit of the Smackover Formation in southern Arkansas, and (iv) inspecting the model for explanatory variable importance and influence. Initial model tuning used the tidymodels framework (*52*) in R (*53*) to test XGBoost, *K*-nearest neighbors, and RF algorithms; RF models consistently had higher accuracy and lower bias, so they were used to train the final model and predict lithium.

### Explanatory variables

Explanatory variables used to tune the RF model included geologic, geochemical, and temperature information for Jurassic and Cretaceous units. The geologic framework of the model domain is expected to influence brine chemistry both spatially and with depth. Explanatory variables used to train the RF model must be mapped across the model domain to create spatially continuous predictions of lithium. Thus, spatially continuous subsurface geologic information is key, although these digital resources are often difficult to acquire. Each sample was attributed with all explanatory variables, regardless of the formation that the sample was collected from. See the Supplemental Materials for details about digitizing and creating model grid–based versions of the explanatory variables.

The final model included 11 explanatory variables: well depth and formation for the brine sample, thickness of the Hosston Formation (*54*–*56*), thickness of the Cotton Valley Formation (*57*–*59*), thickness and facies of the Buckner Member, thickness, altitude of the top, and facies of the Reynolds oolite unit of the Smackover Formation (*27*), bottom-hole temperature of the Smackover Formation (*31*), and H_2_S concentrations in Smackover Formation brines (*4*, *60*). Categorical explanatory variables (facies classes and sample formation) were one hot–encoded (i.e., each category coded as a binary) for the RF model. Explanatory variables were assigned to lithium samples using a Python software (*61*) and the rasterio package (*62*) that extracted the value of the explanatory variable (generally at a 100-m^2^ resolution) to the well location. Explanatory variables were also resampled from the resolution of the source dataset to the 4-km^2^ resolution of the model grid to map predicted lithium.

### RF modeling

RF is a type of ensemble tree machine-learning model where many decision trees are generated using a randomly sampled subset of the explanatory variables to make a prediction (*63*). Predictions for each sample are generated as an average of many model predictions in the ensemble of decision trees (hence, a forest made of trees). RF model tuning, training, and evaluating model performance for predicting lithium concentration were completed in R software (*53*) using the tidymodels framework (*52*).

RF models are tuned by varying hyperparameters that control model structure and accuracy, including the number of randomly selected explanatory variables (mtry), number of trees grown (trees), and the minimum number of observations per node (min_n). The model with the combination of hyperparameters that results in the lowest RMSE between observed and predicted values is considered the most accurate model. Tuning and hyperparameter selection was completed using 80% of the lithium concentration data as a training dataset (*n* = 176) and 10-fold cross-validation. During 10-fold cross-validation tuning, the training data are randomly divided into 10 subsets, and 10% of the data are used as a “testing” dataset to evaluate model performance (based on RMSE). Hyperparameters for RF tuning ranged from 20 to 1000 trees, 3 to 8 mtry, and 2 to 10 min_n for a total of 480 models (*32*). The range of hyperparameters used for model tuning represents a range of values recommended for RF modeling and appropriate for the dataset size. Specifically, a small number of trees was tested to understand how model accuracy changed with the number of trees grown to predict the relatively small sample size of the lithium dataset (*n* = 221).

The most accurate models tend to be the most complex based on the hyperparameters (that is, large trees, large mtry, and small min_n), so a model within one SE of the model with the lowest RMSE was chosen as the final model. As shown in other machine learning predictions of groundwater geochemistry (*64*, *65*), models within one SE of the highest accuracy model (lowest RMSE) are reasonable models to use for predictions. The one-SE models quantify how model predictions vary on the basis of changes in the hyperparameters, which ultimately control model fit and accuracy. The final RF model was trained on the training dataset, and performance was evaluated using the remaining 20% of the data (holdout, *n* = 45).

The final RF model was also inspected to understand which explanatory variables were more important for predicting lithium concentration and how the variables influenced the prediction. Because ensemble tree machine-learning models are inherently complex, a single tree or collection of trees cannot be inspected to directly understand how the algorithm uses explanatory variables to make a prediction. SHAP values were calculated to quantify variable importance and influence using the fastshap (*66*) and shapviz (*67*) packages. For RF models, explanatory variables are generally more important when they are used earlier in tree building or used many times (*19*). SHAP values quantify the additive effect that an explanatory variable has on making each prediction by permutating models through many simulations, thus providing information about how each explanatory variable influences the response prediction (*68*, *69*). In general, more positive SHAP values represent higher magnitude predictions (e.g., greater lithium concentration), and negative SHAP values represent lower magnitude values (e.g., lower lithium concentration).

### Prediction maps

The final trained RF model and resampled grids of explanatory variables were used to make a spatially continuous prediction of lithium concentration at the midpoint altitude of the Reynolds oolite unit of the Smackover Formation. Although the model was trained on brine data from other Jurassic and Cretaceous units, predictions were only made for the Smackover Formation because much of the brine data were collected from the Smackover Formation (89%) and most of the explanatory variables represent conditions in the Smackover Formation. The depth of each cell in the lithium prediction map ranged from approximately 1066 to 3444 m deep over the model domain (*32*).

### RF model uncertainty

Uncertainty in lithium concentration predictions were quantified using one-SE models (*70*). Each combination of hyperparameters from the 425 one-SE models was used to train an RF model and predict lithium across the model domain. At each cell in the model domain, the SD, 5th percentile, 50th percentile (median), and 95th percentile values were calculated from the predicted values from all the one-SE models for that cell. The SD and difference between the 95th and 5th percentiles provide quantitative measures of variability in lithium predictions and, thus, capture model uncertainty. In addition, a qualitative uncertainty was created by comparing the 5th percentile, median, and 95th percentile predictions to a threshold of 100 mg/liter for lithium, which is often cited as the minimum concentration for commercial lithium extraction using current technologies (*7*). If the 5th, median, and 95th percentile predictions at a cell were >100 mg/liter, then the prediction was described as “very likely” > 100 mg/liter and if the 5th, median, and 95th percentile predictions at a cell were ≤100 mg/liter, then the prediction was described as very likely < 100 mg/liter (*32*).

### Lithium resource estimate

The area, thickness, porosity, and water-to-oil ratio of the Smackover Formation were used to calculate brine volume (fig. S2). Much of the oil and brine production occurs within the upper Smackover Formation (*39*, *42*), so the thickness of the Reynolds oolite unit of the upper Smackover Formation from Akin and Graves (*27*) was used as the thickness for brine calculation. Because a porosity map was not available, porosity values from Smackover core plugs (*28*) and field averages (*29*) were used across the model domain. Variation in diagenesis across the south Arkansas shelf is known to control porosity (*30*), and any variation in the porosity values will affect the calculated mass of lithium. A companion US Geological Survey data release provides Python code to evaluate different porosity scenarios for estimating brine volume (*32*). Estimates of water-to-oil ratios for oil reservoirs in southern Arkansas (*29*) were applied to the 4-km^2^ model grid to estimate the volume of pore space that contains water (brine). Where information about oil reservoirs were not present on the basis of source data (*29*), the water-to-oil ratio was assumed to be 0.9 (90% brine) to account for possible small amounts of oil outside of traps. Water-to-oil ratios were multiplied by the area and porosity values to calculate the volume of brine.

The predicted lithium concentration maps from the RF model were multiplied by the calculated brine volume to calculate the mass of lithium in the Reynolds oolite unit of the upper Smackover Formation (fig. S2). By using lithium predictions from the RF model, the mass of lithium can vary spatially across the model domain, which provides a more detailed estimate than assuming an average lithium concentration. In addition, the final RF model and uncertainty bounds (at the 5th and 95th percentile predictions of the one-SE models) were used to provide lower and upper estimates of lithium mass–based RF model uncertainty.

To provide perspective on the mass of lithium from the Smackover Formation that is currently being brought to the surface from oil, gas, or brine production wells, historical brine production data were quantified across the model domain for the past 40 years. Fluid production rates are an important consideration for the viability of lithium extraction from brines (*7*, *71*). Annual water production volumes were acquired from the proprietary Enerdeq datasets for any wells producing from the Smackover Formation in southern Arkansas from 1982 to 2022 (*31*). Annual water volumes were summed for wells within each 4-km^2^ grid cell of the model domain. In each year of extraction, the calculated mass of lithium (based on the volume of brine extracted and the predicted lithium concentration from the RF model) was assumed to be reinjected into the subsurface, as brine injection is used to dispose of wastewater. Therefore, the brine extraction and reinjection are assumed to result in little to no loss of lithium from the brines through time; this includes brines that are stripped of bromine. More research may be warranted to support this assumption because any substantial loss of lithium will affect the calculated mass of lithium in the subsurface and ratio of lithium brought to the surface.

## Supplementary Materials

### This PDF file includes:

Supplementary Text Figs. S1 to S3 Table S1 References
