# Supplementary material for: Evaluation of the lithium resource in the Smackover Formation brines of southern Arkansas using machine learning
Source: Sci Adv. 2024 Sep 27;10(39):eadp8149. doi: 10.1126/sciadv.adp8149 (PMC11430454; doi:10.1126/sciadv.adp8149)
Supplement: Supplementary file 1 — Supplementary Text Figs. S1 to S3 Table S1 References [file sciadv.adp8149_sm.pdf]

Science Advances
AAAS

Supplementary Materials for

Evaluation of the lithium resource in the Smackover Formation brines of southern Arkansas using machine learning

Katherine J. Knierim *et al.*

Corresponding author: Katherine J. Knierim, kknierim@usgs.gov

Sci. Adv. **10**, eadp8149 (2024)
DOI: 10.1126/sciadv.adp8149

This PDF file includes:

Supplementary Text
Figs. S1 to S3
Table S1
References

Disclaimer

Any use of trade, firm, or product names is for descriptive purposes only and does not imply endorsement by the U.S. Government.

Supplementary Text

Explanatory variables used to train the random forest machine-learning model included geologic, geochemical, and temperature information for Jurassic and Cretaceous units of southern Arkansas. Rasters were digitized and converted from vector datasets or created by kriging point data. See the companion U.S. Geological Survey Data Release for datasets *(32)*.

Depth

Depth of the perforated interval from which the brine sample was collected was available for most (97%) of the lithium samples used to train the random forest machine-learning model. Well depths for fifteen wells in the U.S. Geological Survey's Produced Waters Geochemical Database *(4)* were added using associated well metadata (identifiers, location, formation information) and the Arkansas Oil and Gas Commission's Well Logs Cabinet (https://www.aogc.state.ar.us/docuware/welllog.aspx). Where depth was missing, the depth of the middle of the unit from which the sample was collected was used as the depth value in the model. Sample depths ranged from 727 to 3,351 meters below land surface.

Hosston Formation (thickness and altitude)

Thickness and altitude contours of the Lower Cretaceous Hosston Formation *(54-56)* were converted to raster format. The thickness dataset represents the thickness of top of the Lower Cretaceous Travis Peak Formation or Hosston Formation to the top of the Upper Jurassic to Lower Cretaceous Cotton Valley Group. The altitude dataset represents the structural configuration of the top of the Travis Peak Formation or Hosston Formation in feet below sea level. The thickness shapefile was converted to a raster dataset using the ArcPro v3.1 "Feature to Raster" tool (*72*), which creates a raster with the same contour interval as the source dataset (that is, 61 m). The altitude shapefile was converted to a raster dataset using the ArcPro v3.1 "Topo to Raster" tool (*72*), which creates a continuous surface (that is, values vary continuously between contours). The source rasters were created with a 100-m^2 resolution.

Cotton Valley Group (thickness and altitude)

Thickness and altitude contours of the Cotton Valley Group (*57-59*) were converted to a raster format. The thickness dataset represents the thickness of the top of the Cotton Valley Group to the top of the Upper Jurassic Smackover Formation. The altitude dataset represents the structural configuration of the top of the Cotton Valley Group in feet below sea level. The thickness shapefile was converted to a raster dataset using the ArcPro v3.1 "Feature to Raster" tool (*72*), which creates a raster with the same contour interval as the source dataset (that is, 61 m). The altitude shapefile was converted to a raster dataset using the ArcPro v3.1 "Topo to Raster" tool (*72*), which creates a continuous surface (that is, values vary continuously between contours). The source rasters were created with a 100-m^2 resolution.

Buckner Formation (thickness and facies)

The facies and thickness maps of the Upper Jurassic Buckner Formation in southern Arkansas were georeferenced and digitized from Figures 11 and 12, respectively, from Akin and

Graves (*27*). The facies map was converted from vector format to a raster using the following crosswalk: anhydrite/sand/shale = 1, shale = 2, anhydrite = 3. The thickness contours were converted to raster format using the ArcPro "Topo to Raster" tool (*72*), which creates a continuous surface where values vary continuously between contours. The source rasters were created with a 100-m^2 resolution.

Smackover Formation (thickness, altitude, and facies)

The facies, thickness, and altitude maps of the Reynolds oolite unit of the Smackover Formation were georeferenced and digitized from Figures 4, 9, and 10, respectively, from Akin and Graves (*27*). The facies map was converted from vector format to a raster using the following crosswalk: calcarenite-carbonate mudstone = 1, modified grapestone = 2, grapestone = 3, oolite = 4, and interbedded = 5. To ensure the surface for the top of the Smackover Formation extended to the full model domain, regional altitudes from the Gulf Coast were used to extend contours on the southern extent of the model domain (*73*). The thickness and altitude contours were converted to rasters by creating terrains in ArcPro v3.1, which creates a surface that varies continuously between contours (*72*). The source rasters were created with a 100-m^2 resolution.

Smackover Formation Bottom Hole Temperature

The temperature of the Smackover Formation was created by kriging bottom hole temperatures (BHT) collected from wells drilled to the Smackover Formation. BHT were sourced from S&P Global Enerdeq dataset and included 3,270 temperature records from 819 wells across southern Arkansas and northern Louisiana (*31*). Wells ranged in depth from 423 to 4,298 meters deep (median was approximately 2,408 meters deep) and BHT ranged from 27 to 182°C (median was approximately 81°C). It is recommended that BHT be corrected for a given region given a sufficiently large dataset of BHT versus depth because a single BHT value may not reflect conditions in the formation at depth (*74*). However, the kriged BHT surface is used as input to the random forest machine-learning model to predict lithium concentrations, such that BHT needed to be independent of formation depth and, therefore, BHT values were not corrected prior to kriging. Raw BHT values were kriged using the ArcPro v3.1 "Empirical Bayseian Kriging" tool (*72*) with default parameters, which uses multiple simulations to account for error in the semivariogram associated with the input data. The final BHT surface ranged from 54 to 114°C within the model domain. The source raster was created with a 100-m^2 resolution.

Smackover Formation Dissolved Hydrogen Sulfide

Dissolved hydrogen sulfide (H_2S) concentrations in Smackover Formation brines were acquired from the version 3 of the U.S. Geological Survey's Produced Waters Geochemical Database (PWGD) (n = 108) (*4*), collected in southern Arkansas in 2022 (n = 16), or calculated from a U.S. Geological gas concentration dataset (n = 66) (*60*). These data were used to krig a surface representing H_2S in Smackover Formation brines across the model domain.

Aqueous H_2S concentrations were calculated from gas concentrations using H_2S solubility values from Carroll and Mather (*75*), which used Henry's Law to derive H_2S solubility between 0 and 90°C. The temperature for each sample was calculated using the depth of brine collection, assuming a 25°C per km geothermal gradient. Depths ranged from 2.6 to 7.1 km and temperatures from 65 to 177°C. The calculated H_2S solubilities ranged from 1.71^{-3} to 7.40^{-2} mol %. Measured gaseous H_2S values ranged from 0.06 to 49.4 mol %. Based on the sample depths, calculated temperatures, and observed gaseous H_2S concentrations, the calculated dissolved H_2S

ranged from 0.2 to 251.9 ppm. To convert from ppm to mg/L, an average specific gravity value of 1.17 was used, which is the average specific gravity for Smackover Formation brines (*4*). Dissolved H_2S ranged from 0.2 to 294.7 mg/L.

Samples in the PWGD originally from Moldovanyi and Walter (*47*), samples collected in 2022 (*32*), and calculated dissolved H_2S included values less than a 3 mg/L detection limit (i.e., censored values). Three additional censored samples were created at northern extents of the Smackover Formation to ensure that the kriged surface extended across the model domain. H_2S concentrations, not including censored values, ranged from 3 to 670 mg/L collected from depths ranging from approximately 1.3 to 7 km. Based on the minimum observed value of 3 mg/L, censored values were imputed using a detection limit of 3 mg/L and the NADA (*76*) and truncnorm (*77*) packages in R v.4.3 (*39*) to randomly sample over the log distribution of values between 0 and 3 mg/L.

The observed (n = 61) and imputed (n = 121) values were used to krig an H_2S map in ArcPro v3.1 using Empirical Bayesian Kriging, which uses multiple simulations to account for error in the semivariogram associated with the input data (*72*). The Empirical Bayesian Kriging routine used 150 points in each local model, a minimum of 5 points in the search neighborhood, maximum of 15 points in the search neighborhood, and default values for other parameters. H_2S values from the Gulf Coast outside of the model domain were used in kriging to improve accuracy at the model boundaries. The kriged surface ranged from 0 to 647 mg/L, and 0 to 467 mg/L within the model domain. Because of uncertainty in H_2S concentrations, especially at concentrations greater than approximately 100 mg/L, the surface was binned into H_2S classes: <= 3 mg/L was assigned 3 mg/L, > 3 & <= 10 mg/L was assigned 10 mg/L, >10 & <= 50 mg/L was assigned 50 mg/L, >50 & <= 100 mg/L was assigned 100 mg/L, >100 & <=200 was assigned 200 mg/L, >200 & <=300 was assigned 300 mg/L, and >300 mg/L was assigned 467 mg/L (the maximum value).

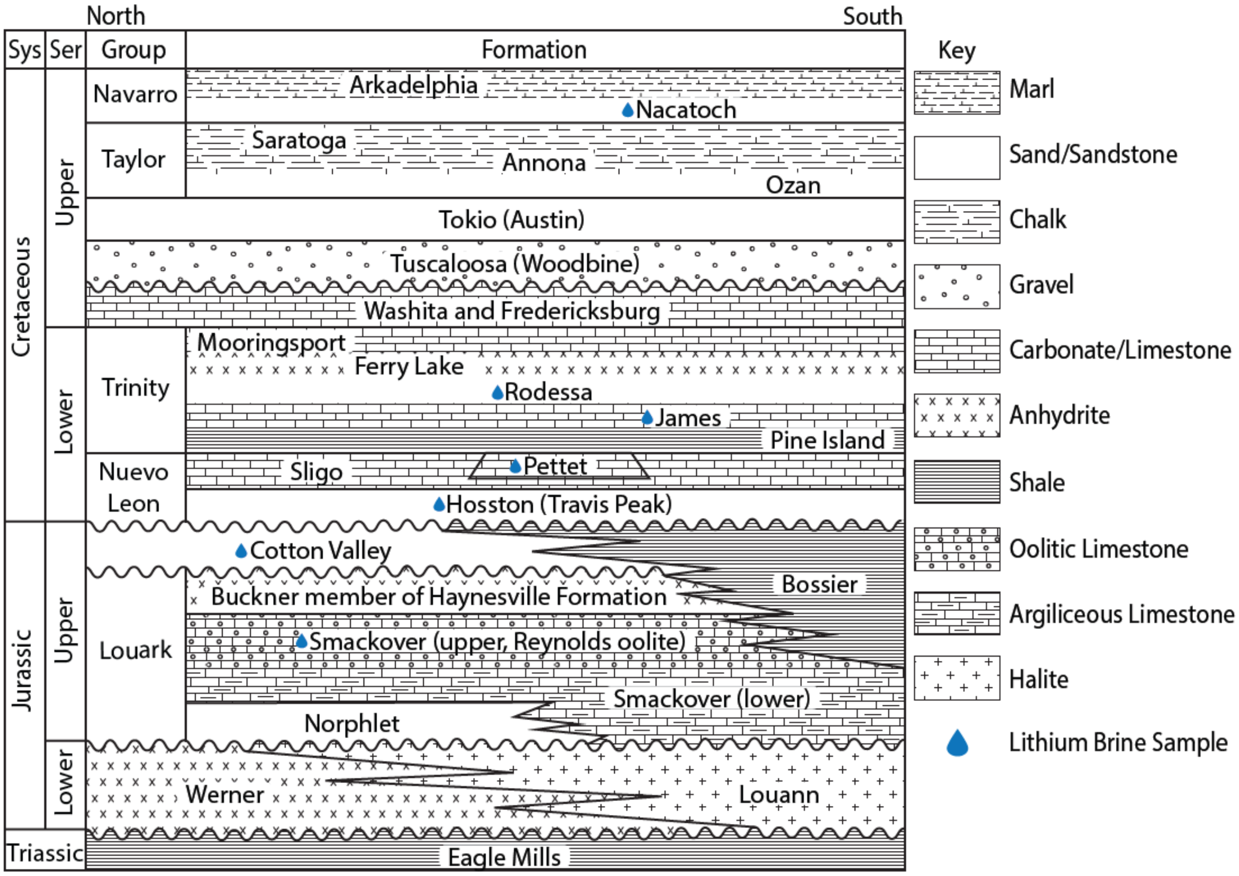

Fig. S1.

Stratigraphic column of geologic units of southern Arkansas showing which units included lithium concentration data from brine samples. For geologic ages and formal formation names, refer to the main text. Modified from (*33*, *34*, *42*, *43*).

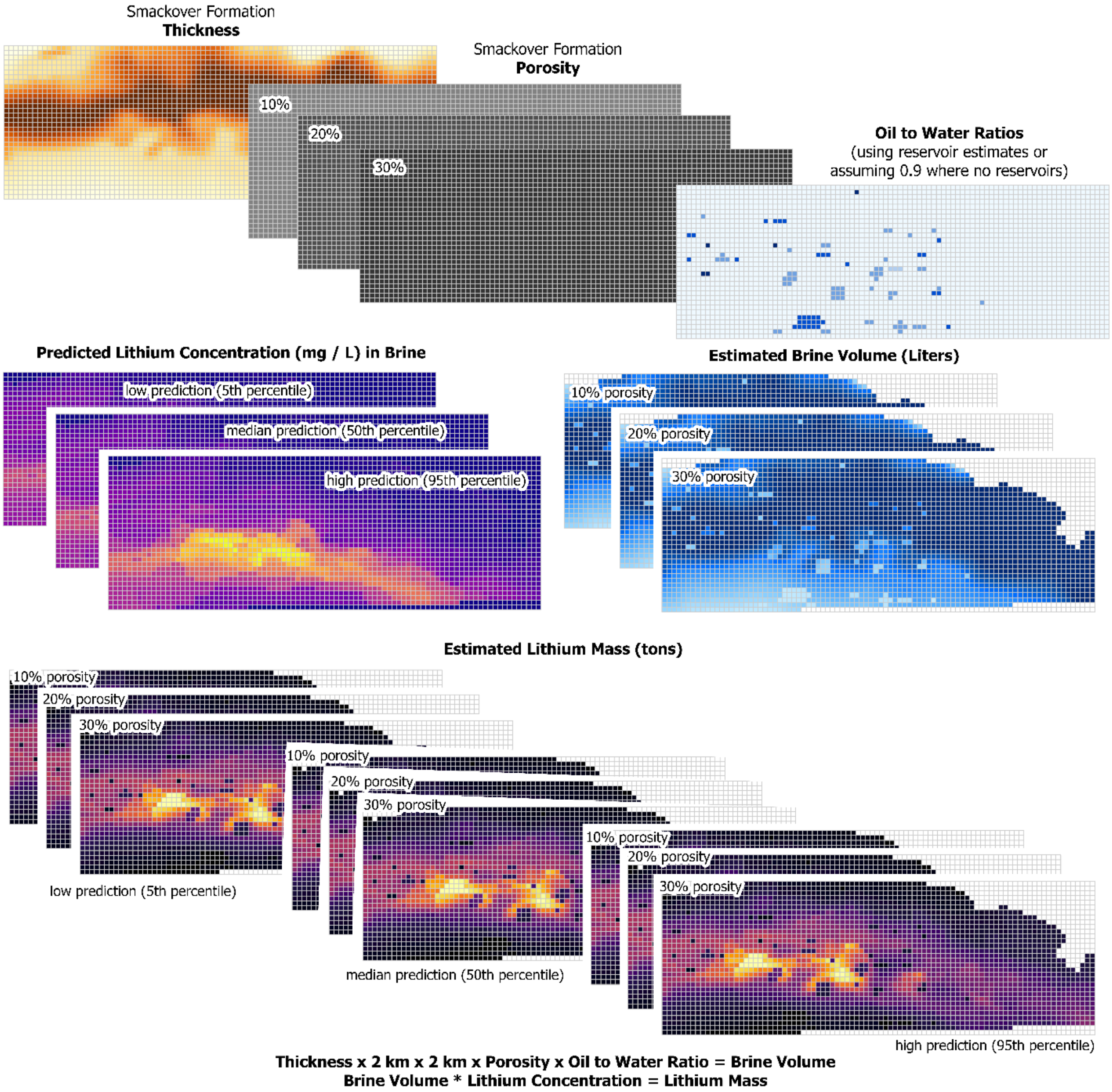

Fig. S2.

Conceptual framework for calculating the mass of lithium in brines in the Smackover Formation in southern Arkansas. Model components represent map views of the model domain.

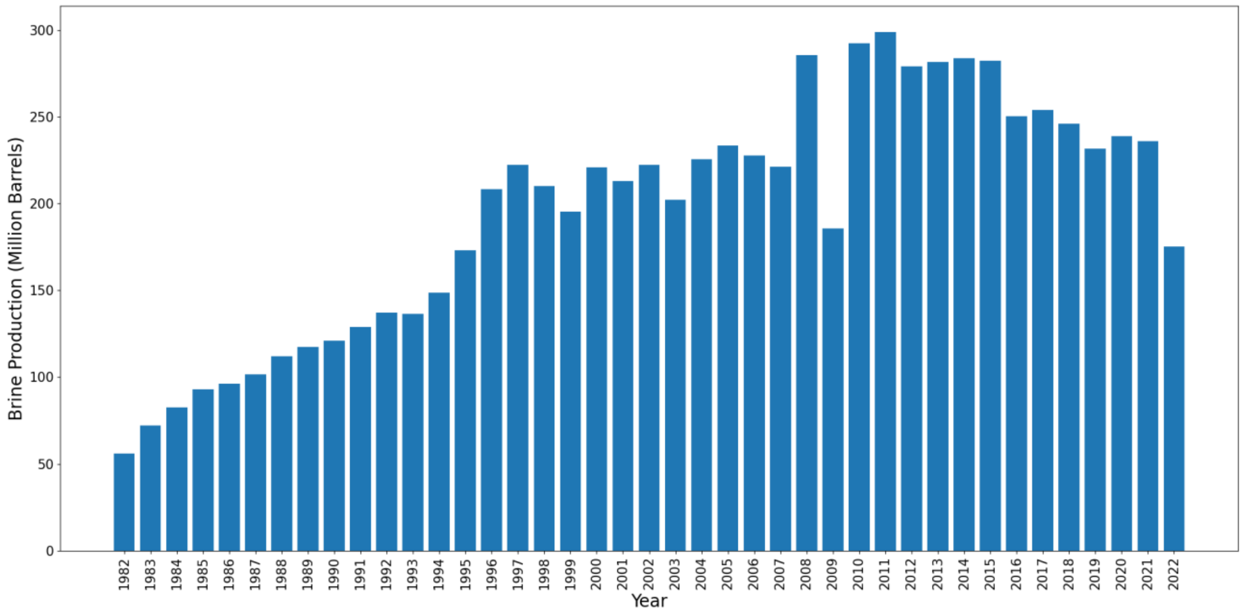

Fig. S3.

Brine production volumes in southern Arkansas from 1982 through 2022 (*27*).

Table S1.

Lithium concentration data for brines in the Smackover Formation and other geologic units in southern Arkansas. For geologic ages and formal formation names, refer to the main text. Data are also available in a companion U.S. Geological Survey data release (*32*).

**Data Source***	**SiteID**	**Simplified Formation**	**Depth (meters)****	**Lithium (mg/L)**
AR2022	SH-1	Cotton Valley	1509	6.26
AR2022	ST-1	Cotton Valley	1737	6.77
PWDB_v3	45877	Cotton Valley	1581	22
AR2022	MP-2	Cotton Valley	2099	22.3
AR2022	DM-7	Cotton Valley	2021	23
PWDB_v3	115212	Cotton Valley	1582	24
AR2022	DM-5-DUP	Cotton Valley	2418	97.4
AR2022	DM-5	Cotton Valley	2418	105
AR2022	DM-6	Cotton Valley	2044	109
AR2022	SM-2	Nacatoch	693	0.654
AR2022	SM-2-DUP	Nacatoch	693	0.665
AR2022	SM-1	Nacatoch	757	4.28
PWDB_v3	115226	Nuevo Leon Grp	1040	5
PWDB_v3	115230	Nuevo Leon Grp	1412	8
PWDB_v3	115225	Nuevo Leon Grp	846	8
PWDB_v3	115231	Nuevo Leon Grp	1402	10
PWDB_v3	115207	Nuevo Leon Grp	1529	10
AR2022	DM-3	Nuevo Leon Grp	1577	15.8
PWDB_v3	115206	Nuevo Leon Grp	1661	23
AR2022	DM-4	Nuevo Leon Grp	-9999	24
PWDB_v3	46091	Smackover	2473	0.27
PWDB_v3	60395	Smackover	3345	0.39
PWDB_v3	45895	Smackover	2137	5
PWDB_v3	45911	Smackover	2246	7
PWDB_v3	115227	Smackover	1512	10
PWDB_v3	17321	Smackover	1355	10
PWDB_v3	17325	Smackover	1340	11
PWDB_v3	17322	Smackover	1330	11
PWDB_v3	17323	Smackover	1385	11
AR2022	SH-2	Smackover	1512	11.7
PWDB_v3	45879	Smackover	1477	12
PWDB_v3	17324	Smackover	1325	12
PWDB_v3	17326	Smackover	-9999	12
PWDB_v3	115233	Smackover	2317	15

*AR2022 = brines collected in 2022 (*32*), PWDB_v3 = historical brine data (*4)*

**Depth below land surface; missing = -9999

Table S1 continued.

Data Source*	SiteID	Simplified Formation	Depth (meters)**	Lithium (mg/L)
PWDB_v3	45880	Smackover	1795	15
PWDB_v3	45881	Smackover	1807	16
PWDB_v3	45878	Smackover	1788	21
PWDB_v3	17328	Smackover	1715	21
PWDB_v3	17327	Smackover	1783	22
PWDB_v3	17329	Smackover	1710	24
PWDB_v3	17330	Smackover	1695	24
PWDB_v3	17296	Smackover	1690	24
PWDB_v3	45876	Smackover	1779	24
PWDB_v3	45348	Smackover	1786	26
PWDB_v3	45916	Smackover	1786	26
PWDB_v3	45917	Smackover	2750	28
PWDB_v3	45875	Smackover	1795	28
PWDB_v3	45887	Smackover	2831	34
PWDB_v3	17295	Smackover	1870	34
PWDB_v3	45897	Smackover	1844	35
PWDB_v3	17294	Smackover	1890	36
PWDB_v3	17310	Smackover	2365	37
PWDB_v3	45915	Smackover	1972	43
PWDB_v3	17337	Smackover	3235	44
PWDB_v3	45884	Smackover	2047	46
PWDB_v3	17331	Smackover	2065	47
PWDB_v3	45896	Smackover	1897	50
PWDB_v3	17333	Smackover	1850	53
PWDB_v3	45912	Smackover	2226	53
AR2022	MW-2	Smackover	2030	54.3
PWDB_v3	46089	Smackover	2182	55
PWDB_v3	115221	Smackover	2120	56
AR2022	MW-1	Smackover	1977	56.7
PWDB_v3	115218	Smackover	2240	59
PWDB_v3	17300	Smackover	1720	59
PWDB_v3	45883	Smackover	2109	62
PWDB_v3	115232	Smackover	2267	64
PWDB_v3	17308	Smackover	2340	64

Table S1 continued.

Data Source*	SiteID	Simplified Formation	Depth (meters)**	Lithium (mg/L)
PWDB_v3	45377	Smackover	1925	66.58
PWDB_v3	17332	Smackover	1980	67
PWDB_v3	17278	Smackover	2115	67
PWDB_v3	115234	Smackover	2268	67
PWDB_v3	115217	Smackover	2225	67
PWDB_v3	45378	Smackover	1934	69.66
PWDB_v3	17289	Smackover	2105	72
PWDB_v3	17315	Smackover	2115	72
PWDB_v3	45885	Smackover	2143	72
PWDB_v3	17335	Smackover	3250	74
PWDB_v3	17277	Smackover	2208	74
PWDB_v3	115220	Smackover	-9999	75
AR2022	BR-3	Smackover	2354	76
PWDB_v3	17276	Smackover	2145	76
PWDB_v3	17319	Smackover	2260	76
PWDB_v3	115219	Smackover	2300	77
PWDB_v3	46090	Smackover	2177	78
PWDB_v3	17309	Smackover	1955	79
PWDB_v3	45874	Smackover	2594	80
PWDB_v3	17314	Smackover	2315	80
PWDB_v3	115222	Smackover	1987	84
AR2022	BR-1	Smackover	2927	84.1
PWDB_v3	17320	Smackover	2280	85
PWDB_v3	45914	Smackover	2193	86
PWDB_v3	17307	Smackover	2005	89
PWDB_v3	17290	Smackover	2070	90
PWDB_v3	45882	Smackover	3333	91
PWDB_v3	115208	Smackover	2423	92
PWDB_v3	17316	Smackover	2135	92
PWDB_v3	45886	Smackover	2005	94
PWDB_v3	17317	Smackover	2120	95
PWDB_v3	115209	Smackover	-9999	95
PWDB_v3	17306	Smackover	1995	95
AR2022	BK-1	Smackover	2173	95.4
PWDB_v3	17336	Smackover	3245	97

Table S1 continued.

Data Source*	SiteID	Simplified Formation	Depth (meters)**	Lithium (mg/L)
PWDB_v3	17311	Smackover	2320	97
PWDB_v3	17304	Smackover	1875	97
PWDB_v3	17305	Smackover	1885	97
PWDB_v3	17313	Smackover	2300	98
PWDB_v3	17312	Smackover	2315	98
AR2022	BK-2	Smackover	2669	98.7
AR2022	BK-2-DUP	Smackover	2669	98.8
PWDB_v3	58226	Smackover	3048	101
PWDB_v3	115235	Smackover	-9999	102
PWDB_v3	17339	Smackover	3055	102
PWDB_v3	17342	Smackover	2755	103
PWDB_v3	17288	Smackover	2180	105
PWDB_v3	17282	Smackover	2190	107
PWDB_v3	17318	Smackover	2115	107
PWDB_v3	17283	Smackover	2195	109
PWDB_v3	17341	Smackover	2765	110
PWDB_v3	17334	Smackover	3225	110
PWDB_v3	17343	Smackover	-9999	111
PWDB_v3	17340	Smackover	3045	111
PWDB_v3	17299	Smackover	2300	115
PWDB_v3	58220	Smackover	3040	116
PWDB_v3	17270	Smackover	2350	116
PWDB_v3	17338	Smackover	3065	117
PWDB_v3	46087	Smackover	2518	118
PWDB_v3	45891	Smackover	2364	122
PWDB_v3	17284	Smackover	2180	122
PWDB_v3	45889	Smackover	2297	125
PWDB_v3	17264	Smackover	2350	126
PWDB_v3	17301	Smackover	1805	126
PWDB_v3	17257	Smackover	2480	132
PWDB_v3	115228	Smackover	2563	134
PWDB_v3	17266	Smackover	2360	136
PWDB_v3	115229	Smackover	2183	136
PWDB_v3	45888	Smackover	2304	140
PWDB_v3	17286	Smackover	2260	144

Table S1 continued.

Data Source*	SiteID	Simplified Formation	Depth (meters)**	Lithium (mg/L)
PWDB_v3	17302	Smackover	2190	145
PWDB_v3	17265	Smackover	2350	149
PWDB_v3	115210	Smackover	2431	159
PWDB_v3	45894	Smackover	2312	160
PWDB_v3	17285	Smackover	2270	162
PWDB_v3	17272	Smackover	2435	165
AR2022	MS-2	Smackover	2284	168
PWDB_v3	17287	Smackover	2392	169
PWDB_v3	45893	Smackover	2352	170
PWDB_v3	115223	Smackover	2437	172
PWDB_v3	17281	Smackover	2499	172
PWDB_v3	115224	Smackover	2469	173
PWDB_v3	115211	Smackover	2404	175
PWDB_v3	17291	Smackover	2245	177
AR2022	BR-6	Smackover	2463	178
PWDB_v3	17293	Smackover	2290	179
PWDB_v3	17292	Smackover	2290	179
PWDB_v3	45892	Smackover	2347	180
PWDB_v3	17279	Smackover	2430	186
PWDB_v3	17256	Smackover	2490	187
PWDB_v3	17280	Smackover	2330	191
PWDB_v3	17303	Smackover	2215	199
AR2022	MS-1	Smackover	2261	207
PWDB_v3	115214	Smackover	2484	214
PWDB_v3	17274	Smackover	2425	223
PWDB_v3	46086	Smackover	2520	225
PWDB_v3	17275	Smackover	2420	227
PWDB_v3	17273	Smackover	2510	228
AR2022	BR-5	Smackover	2713	232
AR2022	BR-2-DUP	Smackover	2652	241
AR2022	BR-4	Smackover	2652	242
PWDB_v3	17268	Smackover	2420	244
AR2022	BR-2	Smackover	2652	252
PWDB_v3	45907	Smackover	2536	260
PWDB_v3	115197	Smackover	2840	265

Table S1 continued.

Data Source*	SiteID	Simplified Formation	Depth (meters)**	Lithium (mg/L)
PWDB_v3	17271	Smackover	2470	269
PWDB_v3	115215	Smackover	2591	269
PWDB_v3	45908	Smackover	2474	276
PWDB_v3	45906	Smackover	2566	277
PWDB_v3	46088	Smackover	2525	282
PWDB_v3	17269	Smackover	2485	288
PWDB_v3	115198	Smackover	2840	302
PWDB_v3	115216	Smackover	2541	302
PWDB_v3	115213	Smackover	2650	312
PWDB_v3	17262	Smackover	2625	316
PWDB_v3	17267	Smackover	2395	327
PWDB_v3	45910	Smackover	2534	329
PWDB_v3	45354	Smackover	2543	331
PWDB_v3	45909	Smackover	2534	331
PWDB_v3	17261	Smackover	2630	338
PWDB_v3	45349	Smackover	2559	340
PWDB_v3	45905	Smackover	2546	343
PWDB_v3	115195	Smackover	2840	347
PWDB_v3	115196	Smackover	2840	352
PWDB_v3	45901	Smackover	2548	357
PWDB_v3	46072	Smackover	2603	358
PWDB_v3	17260	Smackover	2635	364
PWDB_v3	45900	Smackover	2548	365
PWDB_v3	45350	Smackover	2583	367
PWDB_v3	17259	Smackover	2670	370
PWDB_v3	17263	Smackover	2400	371
PWDB_v3	45351	Smackover	2588	378
PWDB_v3	115201	Smackover	2825	380
PWDB_v3	46071	Smackover	2610	382
PWDB_v3	45353	Smackover	2572	386
PWDB_v3	45904	Smackover	2568	391
PWDB_v3	45355	Smackover	2638	403
PWDB_v3	45352	Smackover	2624	412
PWDB_v3	17258	Smackover	2665	423
PWDB_v3	45902	Smackover	2622	425

Table S1 continued.

Data Source*	SiteID	Simplified Formation	Depth (meters)**	Lithium (mg/L)
PWDB_v3	115202	Smackover	2825	425
PWDB_v3	115200	Smackover	2825	439
PWDB_v3	45903	Smackover	2565	440
PWDB_v3	45899	Smackover	2684	445
PWDB_v3	115199	Smackover	2825	461
PWDB_v3	115203	Smackover	2831	465
PWDB_v3	115204	Smackover	2853	467
PWDB_v3	115205	Smackover	2875	477
PWDB_v3	59997	Tokio	947	12
AR2022	MP-1	Trinity Grp	1531	6.86
AR2022	DM-SEP-1	Trinity Grp	1450	9.75
AR2022	DM-2	Trinity Grp	1457	11.1
AR2022	DM-1	Trinity Grp	1450	11.3

REFERENCES AND NOTES

1. D. C. Bradley, L. L. Stillings, B. W. Jaskula, L. Munk, A. D. McCauley, "Chapter K: Lithium" in *Critical Mineral Resources of the United States—Economic and Environmental Geology and Prospects for Future Supply* (US Geological Survey, 2017), vol. 1802, p. 862.

2. US Geological Survey, *Mineral Commodity Summaries 2024* (US Geological Survey, 2024).

3. J. M. Hammarstrom, C. L. Dicken, W. C. Day, A. H. Hofstra, B. J. Drenth, A. K. Shah, A. E. McCafferty, L. G. Woodruff, N. K. Foley, D. A. Ponce, T. P. Frost, L. L. Stillings, "Focus areas for data acquisition for potential domestic resources of 11 critical minerals in the conterminous United States, Hawaii, and Puerto Rico—Aluminum, cobalt, graphite, lithium, niobium, platinum-group elements, rare earth elements, tantalum, tin, titanium, and tungsten" (USGS Numbered Series 2019-1023-B, US Geological Survey, 2020).

4. M. S. Blondes, K. J. Knierim, M. R. Croke, P. A. Freeman, C. Doolan, A. S. Herzberg, J. L. Shelton, *U.S. Geological Survey National Produced Waters Geochemical Database v 3.0, December 2023* (US Geological Survey, 2023).

5. A. G. Collins, *Geochemistry of Anomalous Lithium in Oil-Field Brines* (Geological Survey, Oklahoma, 1978).

6. M. Blois, ExxonMobil plans to use direct lithium extraction in Arkansas, *Chem. Eng. News.* **101**, (2023); https://cen.acs.org/energy/energy-storage-/ExxonMobil-plans-use-direct-lithium/101/i38.

7. E. Knapik, G. Rotko, M. Marszałek, Recovery of lithium from oilfield brines—Current achievements and future perspectives: A mini review. *Energies* **16**, 6628 (2023).

8. E. Bunker, R. Bolton, R. Crossley, M. Broadley, A. Thomas, *Lithium Exploration Tools from Source to Sink* (GeoConvention, 2022); https://geoconvention.com/wp-content/uploads/abstracts/2022/73906-lithium-exploration-tools-from-source-to-sink.pdf.

9. E. J. M. Dugamin, A. Richard, M. Cathelineau, M.-C. Boiron, F. Despinois, A. Brisset, Groundwater in sedimentary basins as potential lithium resource: A global prospective study. *Sci. Rep.* **11**, 21091 (2021).

10. A. Kumar, H. Fukuda, T. A. Hatton, J. H. Lienhard, Lithium recovery from oil and gas produced water: A need for a growing energy industry. *ACS Energy Lett.* **4**, 1471–1474 (2019).

11. International Energy Agency, *Lithium* (International Energy Agency, 2024); www.iea.org/reports/lithium.

12. M. L. Vera, W. R. Torres, C. I. Galli, A. Chagnes, V. Flexer, Environmental impact of direct lithium extraction from brines. *Nat. Rev. Earth Environ.* **4**, 149–165 (2023).

13. J. Mackey, D. J. Bain, G. Lackey, J. Gardiner, D. Gulliver, B. Kutchko, Estimates of lithium mass yields from produced water sourced from the Devonian-aged Marcellus Shale. *Sci. Rep.* **14**, 8813 (2024).

14. B. McDevitt, T. L. Tasker, R. Coyte, M. S. Blondes, B. W. Stewart, R. C. Capo, J. A. Hakala, A. Vengosh, W. D. Burgos, N. R. Warner, Utica/Point Pleasant brine isotopic compositions (δ^{7}Li, δ^{11}B, δ^{138}Ba) elucidate mechanisms of lithium enrichment in the Appalachian Basin. *Sci. Total Environ.* **947**, 174588 (2024).

15. R. Darvari, J.-P. Nicot, B. R. Scanlon, J. R. Kyle, B. A. Elliott, K. Uhlman, Controls on lithium content of oilfield waters in Texas and neighboring states (USA). *J. Geochem. Explor.* **257**, 107363 (2024).

16. G. L. Macpherson, Lithium in fluids from Paleozoic-aged reservoirs, Appalachian Plateau region, USA. *Appl. Geochem.* **60**, 72–77 (2015).

17. E. D. Attanasi, T. C. Coburn, P. A. Freeman, Machine learning approaches to identify lithium concentration in petroleum produced waters. *Miner. Econ.* 10.1007/s13563-023-00409-8, (2024).

18. M. A. Lombard, E. E. Brown, D. M. Saftner, M. M. Arienzo, E. Fuller-Thomson, C. J. Brown, J. D. Ayotte, Estimating lithium concentrations in groundwater used as drinking water for the conterminous United States. *Environ. Sci. Technol.* **58**, 1255–1264 (2024).

19. M. Kuhn, K. Johnson, *Applied Predictive Modeling* (Springer, 2016); https://doi.org/10.1007/978-1-4614-6849-3).

20. K. J. Knierim, J. A. Kingsbury, C. J. Haugh, K. M. Ransom, Using boosted regression tree models to predict salinity in Mississippi Embayment Aquifers, Central United States. *J. Am. Water Resour. Assoc.* **56**, 1010–1029 (2020).

21. E. Farahbakhsh, J. Maughan, R. D. Müller, Prospectivity modelling of critical mineral deposits using a generative adversarial network with oversampling and positive-unlabelled bagging. *Ore Geol. Rev.* **162**, 105665 (2023).

22. J. Podgorski, M. Berg, Global threat of arsenic in groundwater. *Science* **368**, 845–850 (2020).

23. D. R. Eccles, J. Touw, W. Novak, R. M. McGowen, "Maiden inferred bromine- and lithium-brine resource estimations for Tetra Technologies, Inc.'s Tetra Property in Akansas, United States" (S-K 1300 Technical Report, TETRA Technologies, 2022).

24. NORAM Engineering Constructors Ltd., "Standard Lithium LTD. Preliminary economic assessment of SW Arkansas Lithium Project, NI 43-101 Technical Report, amended" (Document No. E3580-RP-0200, NORAM Engineering Constructors Ltd., 2021); www.sec.gov/Archives/edgar/data/1537137/000117184321008188/exh_991.htm.

25. P. Daitch, "Lithium extraction from oilfield brine," thesis, University of Texas, Austin, TX (2018).

26. G. Zhang, Y. Lu, Bias-corrected random forests in regression. *J. Appl. Stat.* **39**, 151–160 (2012).

27. R. H. Akin, R. W. Graves, Reynolds oolite of Southern Arkansas. *Am. Assoc. Pet. Geol.* **53**, 1909–1922 (1969).

28. P. Li, "Core plug analysis of the upper Smackover Formation in Lafayette County, southwestern Arkansas" (Open-File Report 2023–02, Office of the State Geologist, 2023); www.geology.arkansas.gov/publication/open_file_reports/OFR-2023-02-open-file-report.html.

29. Nehring Associates, The significant oil and gas fields of the United States database [data current as of December 2017] (Colorado Springs, 2019).

30. C. H. Moore, Y. Druckman, Burial diagenesis and porosity evolution, Upper Jurassic Smackover, Arkansas and Louisiana. *Am. Assoc. Pet. Geol., Bull.* **65**, 5569412 (1981).

31. S&P Global, Enerdeq US Well History and Production; database available from S&P Global Commodity Insight (2023); www.spglobal.com.

32. K. J. Knierim, M. S. Blondes, P. A. Freeman, A. L. Masterson, B. McDevitt, A. S. Herzberg, C. Doolan, J. M. Chenault, A. Jubb, M. R. Croke, Lithium observations, machine-learning predictions, and mass estimates from the Smackover Formation brines in southern Arkansas (data release, US Geological Survey, 2024); https://doi.org/10.5066/P9QPRYZN.

33. T. Hengl, M. Nussbaum, M. N. Wright, G. B. M. Heuvelink, B. Gräler, Random forest as a generic framework for predictive modeling of spatial and spatio-temporal variables. *PeerJ* **6**, e5518 (2018).

34. US Geological Survey, *Mineral Commodity Summaries 2023* (US Geological Survey, 2023).

35. A. Salvador, "Chapter 8: Triassic-Jurassic" in *The Gulf of Mexico Basin*, A. Salvador, Ed., The Geology of North America (Geological Society of America, 1991), pp. 131–180.

36. K. R. Scott, W. E. Hayes, R. P. Fietz, Geology of the Eagle Mills Formation. *Gulf Coast Assoc. Geol. Soc. Trans.* **11**, 1–14 (1961).

37. J. W. Snedden, W. E. Galloway, *The Gulf of Mexico Sedimentary Basin: Depositional Evolution and Petroleum Applications* (Cambridge Univ. Press, ed. 1, 2019); www.cambridge.org/core/product/identifier/9781108292795/type/book.

38. R. W. Imlay, *Lower Cretaceous and Jurassic Formations of Southern Arkansas and Their Oil and Gas Possibilities*, information circular 12 (Arkansas Resources and Development Commission, Division of Geology, 1940); www.geology.arkansas.gov/docs/pdf/publication/information-circulars/IC-12.pdf.

39. W. B. Weeks, South Arkansas stratigraphy with emphasis on the older coastal plain beds. *AAPG Bull.* **22**, 953–983 (1938).

40. E. A. Mancini, P. Li, D. A. Goddard, R. K. Zimmerman, Petroleum source rocks of the onshore interior salt basins North Central and Northeastern Gulf of Mexico. *Gulf Coast Assoc. Geol. Soc. Trans.* **55**, 486–504 (2005).

41. Arkansas Oil and Gas Commission, Annual report of production 2016 (2016); www.aogc.state.ar.us/annual/default.aspx.

42. J. H. Vestal, *Petroleum Geology of the Smackover Formation of Southern Arkansas*, information circular 14 (Arkansas Geological Survey, 1950); www.geology.arkansas.gov/publication/information-circulars/IC-14-information-circular.html.

43. R. B. Stroud, R. H. Arndt, F. B. Fulkerson, W. G. Diamond, *Mineral Resources and Industries of Arkansas*, bulletin 645 (US Bureau of Mines, 1969); https://digital.library.unt.edu/ark:/67531/metadc12795/m2/1/high_res_d/Bulletin0645.pdf.

44. J. L. Roberts, B. E. Lock, The Rodessa Formation in Bossier Parish Louisiana: Lithofacies analysis of a hydrocarbon productive shallow water clastic-carbonate sequence. *Gulf Coast Assoc. Geol. Soc. Trans.* **38**, 103–111 (1988).

45. S. H. Ogier, *Stratigraphy of the Upper Cretaceous Tokio Formation, Caddo Parish, Louisiana* (Shreveport Geological Society, 1963).

46. T. Cullom, W. Granata, S. Gayer, R. Heffner, S. Pike, L. Hermann, C. Meyertons, G. Sigler, The basin frontiers and limits for exploration in the Cretaceous System of central Louisiana. *Gulf Coast Assoc. Geol. Soc. Trans.* **12**, 97–115 (1962).

47. E. P. Moldovanyi, L. M. Walter, Regional trends in water chemistry, Smackover Formation, Southwest Arkansas: Geochemical and physical controls. *AAPG Bull.* **76**, 864–894 (1992).

48. A. M. Stueber, P. Pushkar, E. A. Hetherington, A strontium isotopic study of Smackover brines and associated solids, southern Arkansas. *Geochim. Cosmochim. Acta* **48**, 1637–1649 (1984).

49. A. G. Collins, *Geochemistry of Liquids, Gases, and Rocks From the Smackover Formation*, report of investigations 7897 (US Bureau of Mines, 1974).

50. M. L. Trout, "Origin of bromide-rich brines in southern Arkansas," thesis, University of Missouri, Columbia, MO (1974).

51. M. S. Blondes, J. L. Shelton, M. A. Engle, J. P. Trembly, C. A. Doolan, A. M. Jubb, J. C. Chenault, E. L. Rowan, R. J. Haefner, B. E. Mailot, Utica shale play oil and gas brines: Geochemistry and factors influencing wastewater management. *Environ. Sci. Technol.* **54**, 13917–13925 (2020).

52. M. Kuhn, H. Wickham, Tidymodels: A collection of packages for modeling and machine learning using tidyverse principles, version 1.1 (2020); www.tidymodels.org.

53. R Core Team, R: A language and environment for statistical computing, version 4.3, R Foundation for Statistical Computing (2023); www.r-project.org/.

54. T. S. Dyman, S. M. Condon, *Estimated Thickness of the Travis Peak-Hosston Formations to the Top of the Cotton Valley Group, Western Gulf and East Texas Basin and Louisiana-Mississippi Salt Basins Provinces (047, 048 and 049)* (US Geological Survey, 2005).

55. T. S. Dyman, S. M. Condon, *Structure Contour of the Top of the Travis Peak-Hosston Formations, Western Gulf and East Texas Basin and Louisiana-Mississippi Salt Basins Provinces (047, 048 and 049)* (US Geological Survey, 2005).

56. T. S. Dyman, S. M. Condon, "Chapter 5: Assessment of undiscovered conventional oil and gas resources—Lower Cretaceous Travis Peak and Hosston Formations, Jurassic Smackover interior salt basins total petroleum system, in the East Texas Basin and Louisiana-Mississippi Salt Basins provinces" in *Petroleum Systems and Geologic Assessment of Undiscovered Oil and Gas, Cotton Valley Group and Travis Peak-Hosston Formations, East Texas Basin and Louisiana-Mississippi Salt Basins Provinces of the Northern Gulf Coast Region (Data Series 69-E)*, Digital Data Series (US Geological Survey, 2006), p. 43; https://doi.org/10.3133/ds69E_chapter5.

57. T. S. Dyman, S. M. Condon, "Chapter 2: Assessment of undiscovered conventional oil and gas resources—Upper Jurassic–Lower Cretaceous Cotton Valley Group, Jurassic Smackover interior salt basins total petroleum system, in the East Texas Basin and Louisiana-Mississippi Salt Basins provinces" in *Petroleum Systems and Geologic Assessment of Undiscovered Oil and Gas, Cotton Valley Group and Travis Peak–Hosston Formations, East Texas Basin and Louisiana-Mississippi Salt Basins Provinces of the Northern Gulf Coast Region*, Digital Data Series (US Geological Survey, 2006), p. 52; https://doi.org/10.3133/ds69E_chapter2.

58. T. S. Dyman, S. M. Condon, "*Estimated Thickness of the Cotton Valley Group to the Top of the Smackover Formation, Western Gulf and East Texas Basin and Louisiana-Mississippi Salt Basins Provinces (047, 048 and 049)*" (US Geological Survey, 2005).

59. T. S. Dyman, S. M. Condon, *Structure Contour of the Top of the Cotton Valley Group, Western Gulf and East Texas Basin and Louisiana-Mississippi Salt Basins Provinces (047, 048 and 049)* (US Geological Survey, 2005).

60. S. T. Brennan, J. L. Rivera, B. Varela, A. J. Park, L. A. Agyepong, *Natural Gas Compositional Analyses Dataset of Gases from United States Wells* (US Geological Survey, 2021).

61. Python Software Foundation, Python language reference, v 3.9, Python.org (2023); www.python.org.

62. S. Gillies, Rasterio: Geospatial raster I/O for {Python} programmers, version 1.2, Mapbox (2023); https://github.com/rasterio/rasterio.

63. L. Breiman, Random forests. *Mach. Learn.* **45**, 5–32 (2001).

64. K. J. Knierim, J. A. Kingsbury, K. Belitz, P. E. Stackelberg, B. J. Minsley, J. R. Rigby, Mapped predictions of manganese and arsenic in an alluvial aquifer using boosted regression trees. *Groundwater* **60**, 362–376 (2022).

65. B. T. Nolan, M. N. Fienen, D. L. Lorenz, A statistical learning framework for groundwater nitrate models of the Central Valley, California, USA. *J. Hydrol.* **531** (Pt. 3), 902–911 (2015).

66. B. M. Greenwell, fastshap: Fast approximate Shapley values, version 0.1 (2023); https://github.com/bgreenwell/fastshap.

67. M. Mayer, A. Stando, shapviz: SHAP visualizations, version 0.9 (2023); https://cran.r-project.org/web/packages/shapviz/index.html.

68. S. M. Lundberg, S.-I. Lee, "A unified approach to interpreting model predictions" in *31st Conference on Neural Information Processing Systems (NIPS 2017)* (Advances in Neural Information Processing Systems 30, 2017); https://proceedings.neurips.cc/paper_files/paper/2017/file/8a20a8621978632d76c43dfd28b67767-Paper.pdf.

69. S. M. Lundberg, G. G. Erion, S.-I. Lee, Consistent individualized feature attribution for tree ensembles. arXiv:1802.03888 [cs.LG] (7 March 2019).

70. C. D. Killian, K. J. Knierim, "Machine-learning predictions of groundwater specific conductance in the Mississippi Alluvial Plain, South-Central United States, with evaluation of regional geophysical aerial electromagnetic data as explanatory variables" (Scientific Investigations Report 2023–5099, US Geological Survey, 2023).

71. M. Marza, G. Ferguson, J. Thorson, I. Barton, J.-H. Kim, L. Ma, J. McIntosh, Geological controls on lithium production from basinal brines across North America. *J. Geochem. Explor.* **257**, 107383 (2024).

72. Esri, ArcGIS Pro, version 3.1 (2023); www.esri.com/en-us/arcgis/products/arcgis-pro/overview.

73. American Association of Petroleum Geologists, Reservoirs and Petroleum Systems of the Gulf Coast (2023); www.datapages.com/gis-map-publishing-program/gis-open-files/geographic/reservoirs-and-petroleum-systems-of-the-gulf-coast.

74. L. A. Burke, O. N. Pearson, S. A. Kinney, "New method for correcting bottomhole temperatures acquired from wireline logging measurements and calibrated for the Onshore Gulf of Mexico Basin, U.S.A." (Open-File Report 2019–1143, US Geological Survey, 2019).

75. J. J. Carroll, A. E. Mather, The solubility of hydrogen sulphide in water from 0 to 90°C and pressures to 1 MPa. *Geochim. Cosmochim. Acta* **53**, 1163–1170 (1989).

76. L. Lee, NADA package, version 1.6 (2020); https://CRAN.R-project.org/package=NADA.

77. O. Mersmann, H. Trautmann, D. Steuer, B. Bornkamp, truncnorm: Truncated normal distribution, version 1.0 (2023); https://cran.r-project.org/web/packages/truncnorm/index.html.
